# Assessment of Online Adaptive MRI-Guided Stereotactic Body Radiotherapy (SBRT) With an Integrated Boost to the Dominant Lesion in Prostate Cancer

**DOI:** 10.7759/cureus.94227

**Published:** 2025-10-09

**Authors:** Kyle R Padgett, Jonathan Cabrera, Levent Sensoy, William Jin, Matthew C Abramowitz, Matthew Studenski, Hayden Guerrero, Alberto de la Zerda, Siamak P Nejad-Davarani, John C Ford, Nesrin Dogan

**Affiliations:** 1 Department of Radiation Oncology, Sylvester Comprehensive Cancer Center, Miller School of Medicine, University of Miami, Miami, USA; 2 Department of Radiology, Miller School of Medicine, University of Miami, Miami, USA; 3 Department of Biomedical Engineering, University of Miami, Miami, USA

**Keywords:** adaptive radiation therapy, external beam radiation, magnetic resonance imaging, mri guided radiation therapy, prostate cancer, simultaneous integrated boost, stereotactic body radiotherapy

## Abstract

Purpose/objective

Online adaptive radiotherapy (ART) with daily MR imaging can improve dosimetric accuracy by accounting for anatomical changes throughout the course of treatment. Existing daily ART prostate studies have diverse conclusions, but most of these studies do not include simultaneous integrated boosts (SIBs) to the dominant intraprostatic lesion, which has shown an increase in biochemical disease-free survival. The purpose of this study is to evaluate the potential dosimetric benefits of daily ART with SIB to the dominant intraprostatic lesion and other targets, as well as the potential sparing of organs-at-risk (OARs) for prostate cancer, and to compare these results with those reported in other published studies.

Materials/methods

Thirteen patients with prostate cancer treated with MR-guided stereotactic body radiotherapy (SBRT) were included in this study. The prescribed dose to the planning target volume (PTV) was 36.25 Gy in five fractions. The prescription to the intraprostatic boost to the gross tumor volume (GTV) ranged between 40 and 45 Gy. All SBRT fractions employed daily MR-guided setup, and OARs included the bladder, anorectum, bowel, femoral heads, and penile bulb. Daily MRIs and associated contours were used to create adapted treatment plans. For each simulated adapted fraction, the plan was re-optimized based on the contours from the daily setup MRI. Dosimetric metrics for non-adaptive fractions were simulated by re-calculating the dose on each daily setup MRI. PTV and GTV coverage and OAR constraints were used to compare non-adaptive and adaptive approaches.

Results

PTV coverage at the prescription dose level exceeded 90% in 35 out of 64 non-adapted treatment fractions (55%). Conversely, 29 fractions (45%) had less than 90% coverage, with 12 fractions (19%) showing coverage below 85%. The clinical target volume (CTV) coverage was greater than 95% in 52/64 fractions and was below 90% coverage in only 1/64 fractions (1.5%). GTV coverage at the boost dose level ranged from 55% to 100%, with 38 out of 74 fractions (51%) achieving 90% or greater coverage at the prescribed dose. A substantial number of fractions 27/74 (36%) had coverage below 85%, with a handful of fractions exhibiting less than 70% coverage, 5/74 fractions or 7%. All adapted fractions met prescribed coverage for the GTV, CTV, and PTV. Additionally, OAR constraint violations were evaluated and compared for the bladder and anorectum. Very few OAR constraints were exceeded in the non-adaptive setting, and none were exceeded in the adaptive setting. The anorectum dose constraint was exceeded in 22% of the non-adaptive fractions, while all adaptive fractions met the prescribed constraint. All non-adaptive and adaptive fractions met the prescribed bladder dose constraints; however, the adaptive fractions demonstrated some degree of bladder sparing compared to the non-adaptive fractions. Accumulated dose volume histograms were also generated for each patient to evaluate cumulative differences in target coverage and OAR sparing.

Conclusion

Online adaptive MR-guided SBRT for prostate cancer, utilizing daily plan re-optimization, resulted in improved target conformality, enhanced coverage, and increased OAR sparing compared to non-adaptive SBRT. The primary benefit observed was improved GTV coverage. Daily adaptive planning enables satisfactory GTV coverage without the need for added margins, while also providing better OAR sparing.

## Introduction

MRI-guided radiotherapy (MRgRT) systems can account for both intra-fraction and inter-fraction changes by utilizing three-dimensional MR imaging, on-table adaptive treatment planning, and gating of radiation treatment with real-time MR imaging [1-4]. MRgRT systems enable a level of soft tissue tracking and selective radiation delivery not previously available. The ability to better see the prostate as well as organs at risk (OARs) provides an opportunity to re-optimize the daily treatment plan to account for differences in target and OAR size, position, and shape. The potential benefit of on-table adaptive treatment planning is most fully appreciated for treatment sites where targets and/or OARs have a significant amount of inter-fraction motion or where the OAR volume may drastically change [5]. Dosimetric adaptive studies are particularly important for sites such as prostate cancer, where the target volume can be located in close proximity to sensitive organs such as the bladder and the rectum, and where the filling level of these OARs can vary significantly. MRI-guided systems may have advantages over cone-beam CT (CBCT)-guided systems where CBCT imaging may be poor at defining both the targets and the OARs with limited low contrast resolution. On-table adaptive approaches require considerable time for planning and delivery, adding significant complexity to stereotactic body radiotherapy (SBRT) treatments [6-8]. Dosimetric studies like the present one help to identify which disease sites derive the greatest benefit from these adaptive techniques.

Treating prostate cancer with SBRT is now an established approach rather than an emerging technique [9-11]. The most common prescription doses for prostate SBRT are 36.25 Gy and 40 Gy delivered in five fractions. The most frequent side-effects associated with prostate SBRT are related to GI symptoms, urinary symptoms, and sexual function [12]. Some studies also incorporate a simultaneous integrated boost (SIB) to the dominant prostatic lesion(s) to potentially improve local control and reduce recurrence [13,14]. However, there is currently no consensus in the literature regarding the necessity or appropriate margin size for these gross tumor volume (GTV) targets. Reported margin expansions have ranged from 0 to 5 mm [15,16]. Successful SBRT of the prostate relies on accurate image-guided radiotherapy (IGRT) to administer adequate dose to the target while minimizing dose to the relevant OARs. Conventional IGRT approaches often include on-board cone-beam computed tomography (CBCT), but due to inherently low contrast between the prostate and the surrounding soft tissue, CT-based IGRT may result in suboptimal image guidance [17]. However, fiducials, typically gold, implanted within the prostate can also be used to aid in the alignment of patients when utilizing CT-based IGRT. MRI guidance provides superior setup imaging as compared to CBCT guidance which allows for better targeting of the prostate and other targets and delivers better visualization of the whole pelvis including the OARs. This improved visualization of the OARs and surrounding anatomy facilitates accurate adaptive treatment planning to improve target coverage and OAR sparing [18].

With the adoption of MRgRT systems, a handful of publications have highlighted their benefits for delivering SBRT to the prostate. Several studies have specifically focused on the safety and feasibility of delivering prostate MRI-guided adaptive radiation therapy (MRgART) SBRT treatments. These studies primarily focused on prostate-specific antigen (PSA) metrics, toxicity profiles, and quality-of-life (QoL) assessments. Overall, they demonstrate that MRgART treatments are safe, comparable to or in some cases better than conventional IGRT, and effective in disease control [19-22]. A notable study by Nicosia et al. [23] used dosimetric comparisons to evaluate the effectiveness of MRgART versus conventional IGRT treatment in two patient groups: those with and without implanted fiducials. The study demonstrated the significant dosimetric benefits of MRgART over IGRT in prostate patients without fiducials, while the benefits were more modest in patients treated with fiducial-based IGRT [23].

Other studies have explored the potential advantages of MRgART compared to non-adaptive MRgRT, but direct dosimetric comparisons between adaptive and non-adaptive approaches were not performed. Bohoudi et al. [24] employed deformable dose accumulation across all fractions of an MRgART treatment course and found that higher cumulative bladder doses were associated with increased urinary symptoms. They proposed using mid-course dose accumulation as a strategy to identify patients who may benefit from an updated treatment plan to improve bladder sparing [24]. Dassen et al. [8] analyzed three common approaches to prostate SBRT treatments using the Unity MR-Linac system (Elekta, Stockholm, Sweden): adapt to shape, adapt to rotation, and full adaptive planning. Their study examined how each approach influences the treatment margins required and demonstrated that larger margins can increase the overlap between target volumes and OARs, potentially compromising OAR sparing [8]. One study in particular by Leeman et al. directly investigates the dosimetric benefits of MRgART compared to non-adaptive MRgRT, evaluating both target coverage and OAR sparing [25]. This is a well-executed study that provides valuable evidence supporting the benefits of adaptive treatments. The target dosimetric comparisons made in this study focus on the planning target volume (PTV); no GTV targets were utilized and clinical target volume (CTV) comparisons were not made. Evaluating CTV coverage may provide a more meaningful assessment of whether the clinical treatment goals are achieved, as the PTV is primarily used to account for daily variations, while the clinical objective is to treat the CTV to full dose. Additionally, this study did not include an SIB target volume, which is increasingly common in prostate SBRT treatments. A study published by Brennan et al. [26] does include an SIB target for dose escalation and evaluates the margin requirements needed to account for intra-fraction motion during MRgART treatments. However, it does not compare the dosimetric differences between adaptive and non-adaptive strategies.

In this study, 13 prostate cancer patients treated with SBRT on an MRgRT system (MRIdian, ViewRay Systems Inc., Oakwood Village, OH) were evaluated to investigate potential gains in OAR sparing and coverage metrics for the GTV and CTV when using adaptive versus non-adaptive treatment approaches. The direct dosimetric comparisons of GTV and CTV coverage provide new evidence supporting the potential advantages of daily adaptation, while the OAR findings contribute to the limited but growing body of literature that quantitatively compares adaptive and non-adaptive strategies. Together with previous studies, the results presented here and future investigations will help clarify whether MRgART offers significant clinical benefits warranting broader adoption or if its advantages are modest and better suited for selective use. This article was previously presented as a meeting abstract at the 2022 ESTRO Annual Scientific Meeting in May 2022.

## Materials and methods

Patient and treatment characteristics

Thirteen prostate cancer patients enrolled in an IRB-approved study and treated with MRgRT were included in this study. This study is approved by the University of Miami Human Subject Research Office, Miami, USA (approval number: 20160817, dated: 15-01-2025). The ages of these patients ranged from 56 to 83 years, with an average age of 70. These patients were treated at the University of Miami Sylvester Comprehensive Cancer Center between May 2020 and June 2022. The subject inclusion criteria included age of at least 18 years, histologically confirmed prostate adenocarcinoma, and multi-parametric MRI (mpMRI)-defined dominant lesion/s. The exclusion criteria included prior prostatectomy, prior pelvic radiotherapy, bilateral hip prostheses, and inability to undergo MRI. Table 1 summarizes the patient population information: age, target volumes, prescription doses, GTV coverage constraints, and PTV coverage constraints.

**Table 1:** Summary of the patient population: age, target volumes, prescription doses, and coverage constraints. This table summarizes the patient population information: age, target volumes, prescription (Rx) doses, GTV coverage constraints and PTV coverage constraints. ♦: Patient #12 had three gross tumor volume (GTV) targets, the value of 5 cm^3^ reported in the table is the sum of all three GTVs. ▪: Patient #10 was treated with a simultaneous integrated boost (SIB) GTV target for four out of five fractions. Due to increasing urinary symptoms, the GTV target was discontinued for the fifth fraction. Therefore, this fraction was removed from analyses for both adaptive and non-adaptive scenarios. PTV: planning target volume.

Patient #	1	2	3	4	5	6	7	8	9	10	11	12	13
Age	69	77	60	61	79	83	75	75	78	59	67	74	56
GTV volume (cc)	1.5	1.0	3.0	11.7	1.8	3.7	2.5	2.1	13.4	1.3	3.4	5.0 ♦	1.4
PTV volume (cc)	91.8	126.8	102.3	133.9	112.6	147.8	127.9	155.0	267.1	165.3	173.8	115.3	91.8
GTV Rx (Gy)	40	40	40	45	40	40	40	40	45	40	42	42	40
PTV Rx (Gy)	36.25	36.25	36.25	36.25	36.25	36.25	36.25	36.25	36.25	36.25	36.25	36.25	36.25
# of fractions	5	5	5	5	5	5	5	5	5	5 ▪	5	5	5
GTV dose per Fx (Gy)	8	8	8	9	8	8	8	8	9	8	8.4	8.4	8
PTV dose per Fx (Gy)	7.25	7.25	7.25	7.25	7.25	7.25	7.25	7.25	7.25	7.25	7.25	7.25	7.25
GTV coverage constraints	90%	90%	90%	90%	90%	90%	95%	90%	90%	90%	90%	90%	90%
PTV coverage constraints	95%	96%	95%	90%	95%	95%	95%	95%	95%	95%	95%	95%	95%

Simulation and contouring

On the day of simulation, patients underwent both CT and MRI imaging studies in the treatment position, with the second study typically acquired immediately after the first one, usually, the CT simulation was performed first. The MRI simulation consisted of a 3D true fast imaging with steady-state precession (FISP) sequence acquired in the head-first supine position. The MRgRT system has several predefined imaging protocols available for pelvic imaging. For each patient, the scan with the most appropriate field of view (FOV) was selected to maximize image quality. A commonly selected protocol was a true-FISP scan with the following parameters: time of repetition/time of echo (TR/TE): 3.3/1.4ms, flip angle: 60°, bandwidth: 537 Hz/pixel, FOV: 400x400x432 mm^3^, imaging matrix: 266x266x144, and voxel size: 1.5x1.5x3 mm^3^. The spatial integrity of the imaging system is assessed monthly, with distortions measuring less than 1 mm within a 20-cm-diameter sphere and less than 2 mm within a 35-cm-diameter sphere. The MRI dataset collected on the MRgRT system served as the primary planning dataset, while the planning CT with 2 mm slice thickness was used for electron density information. The planning CT study was deformably registered to the planning MRI dataset utilizing the deformable algorithm available in the Viewray TPS (MRIdian, ViewRay Systems Inc., Oakwood Village, OH). The deformable registration was chosen over rigid to ensure that the electron density information was mapped effectively to the planning MRI with minimal discrepancies. Since the planning CT and planning MRI studies were collected within one hour of each other utilizing the same patient positioning methods, the deformations needed were quite small and resulted in quality registrations that were assessed by physics at the time of treatment planning to ensure accurate dose calculations. All patients were imaged in the supine head-first position with their hands on their chest and with a #7 leg-immobilization cushion from Vanarsdale (Vanarsdale Innovative Products Inc., Pensacola, FL) to reproducibly position their legs. All patients followed the same bladder and bowel preparation protocol, maintaining an empty rectum and full bladder, and identical IGRT strategies were applied.

All target and OAR contouring are completed by radiation oncologists who specialize in the genitourinary (GU) treatment site. The GTV is created by incorporating the dominant lesion(s) identified from a multi-parametric MRI (mpMRI), collected on a 3T MRI system. These lesions are determined from several acquisitions from the mpMRI: the early-phase contrast enhancement using a dynamic contrast enhancement (DCE) dataset, water restriction on apparent diffusion coefficient (ADC) maps, and abnormalities on T2-weighted imaging [27]. The mpMRI is rigidly registered to the planning MRI to guide accurate contouring of the GTV target(s) on the planning MRI. This registration was performed by a medical physicist with significant experience performing GU MRI registrations and the registration focused on matching the area of dominant lesion within the prostate to the planning MRI. The alignment of each sequence of the mpMRI to the planning MRI was assessed for accuracy by the physicist to ensure accurate GTV contouring. In this study, only the prostate, proximal seminal vesicles, and dominant intra-prostatic lesion(s) were included as targets; no pelvic lymph nodes were treated. The clinical target volume (CTV) consisted of the prostate and the proximal seminal vesicles. The GTV target did not employ a margin, the PTV was generated by expanding the CTV by 5 mm in all directions except posteriorly, where a 3 mm margin was applied. OARs contoured for treatment planning included the anorectum, bowel, bladder, penile bulb, and femoral heads.

The prescribed dose to the PTV was 36.25 Gy delivered in five fractions, normalized to cover 90%-96% of the PTV volume receiving the full prescribed dose. A 95% normalization was applied in 11 of the 13 patients; one patient received 96% normalization, and one received 90% normalization. The prescribed dose to the GTV(s), defined as the dominant intraprostatic lesion(s), was either 40, 42, or 45 Gy delivered using SIB approach. No additional margin was applied to the GTV(s). Table 1 provides detailed prescription doses to both PTV and GTV targets as well as normalization utilized.

MRgRT system and treatment planning

The MRgRT system (MRIdian, ViewRay Systems Inc., Oakwood Village, OH) integrates a 0.35T MRI scanner with a dedicated 6 mega voltage (MV) flattening filter-free (FFF) linear accelerator. The system is well described in the article by Klüter [28]. Patient data including the planning MRI, planning CT images, and contours for the GTV(s), CTV, PTV, and OARs were imported into the treatment planning system (TPS). The initial “step-and-shoot” intensity modulated radiation therapy (IMRT) plans utilized the planning MRI as the primary dataset and used a single-isocenter and 13-31 beam angles, selected to avoid any beam passing through the couch edges. Monte Carlo dose calculations were performed using a 2 mm dose-grid resolution. Additionally, all treatment plans accounted for the influence of the 0.35T magnetic field on the radiation dose distribution.

Simulated adaptive treatment plans were generated using the fractional MRI datasets acquired for treatment setup and delivery. The OAR structures and the electron density information from the initial treatment plan were deformably registered to the daily MRI. The appropriateness of the electron density map was reviewed by the physicist and could be corrected with contour overrides if deemed appropriate, this was rarely necessary. The prostate and proximal seminal vesicles were manually recontoured on each fractional MRI, by a radiation oncologist, rather than deformably propagated. The PTV was subsequently generated based on these updated structures. The GTV target was migrated from the initial planning MRI dataset to the fractional MRI dataset utilizing a rigid registration that was optimized to the region of the prostate where the GTV target is located. The deformed OAR structures were reviewed and edited by a radiation oncologist prior to plan optimization or dose calculation. Subsequently, the initial treatment plan was re-optimized to match the daily anatomy using the same beam angles and optimization constraints.

To generate the non-adaptive treatment plans, the initial treatment plan was transferred to the fractional MRI datasets using the same alignment employed for treatment delivery. The contours and electron density map previously edited by the physicist and physician for the adaptive treatment planning process were also transferred to the daily MRI dataset. Once these steps were completed, the initial plan was recalculated on the daily anatomy and compared to the corresponding simulated adaptive treatment plans.

Analysis methods

Evaluation of the non-adapted and adapted plans was performed by comparing constraint performance between the two planning approaches, analyzing dose volume histograms (DVHs), and conducting qualitative reviews of isodose line distributions. Dose constraints and the doses achieved by both plan types for OARs, GTV(s), CTV, and PTV for all fractions were extracted from the TPS and recorded. Dose values were normalized by their respective constraints for each structure and patient to enable inter-patient comparisons. Although the same prescription and OAR constraints were generally employed, some patients deviated on one or more constraints. This normalization approach provides a consistent framework for comparing all patients.

To compare the impact of adaptation within each patient over the entire treatment course, an average DVH was created by exporting the tabulated DVH data for targets and OARs from each fraction and then calculating the mean across all fractions. Average DVHs for the anorectum and bladder are shown in the Results section for selected patients. The y-axis represents the percentage volume of the target and normal organs, while the x-axis shows the absolute dose. The limitations of this approach are described in the Discussion section. To visually illustrate the impact of adaptation on individual fractions, isodose lines (IDLs) are also included in the Results section. The patients and fractions presented were selected based on noticeable differences between adaptive and non-adaptive treatment plans. Targets, OARs, and relevant isodose levels were displayed on daily images for both non-adapted and adapted plans.

## Results

Coverage constraints

In total, 64 non-adaptive fractions and 64 simulated adaptive fractions from 13 patients were analyzed. Due to the nature of the adaptive process, all 64 fractions in the adaptive cohort were reoptimized and normalized to the prescribed dose, ensuring that GTV, CTV, and PTV coverage constraints were met. For GTV coverage, while there were 64 non-adaptive fractions analyzed, one patient had three GTV targets, resulting in 74 total opportunities to evaluate GTV coverage.

**Figure 1:**
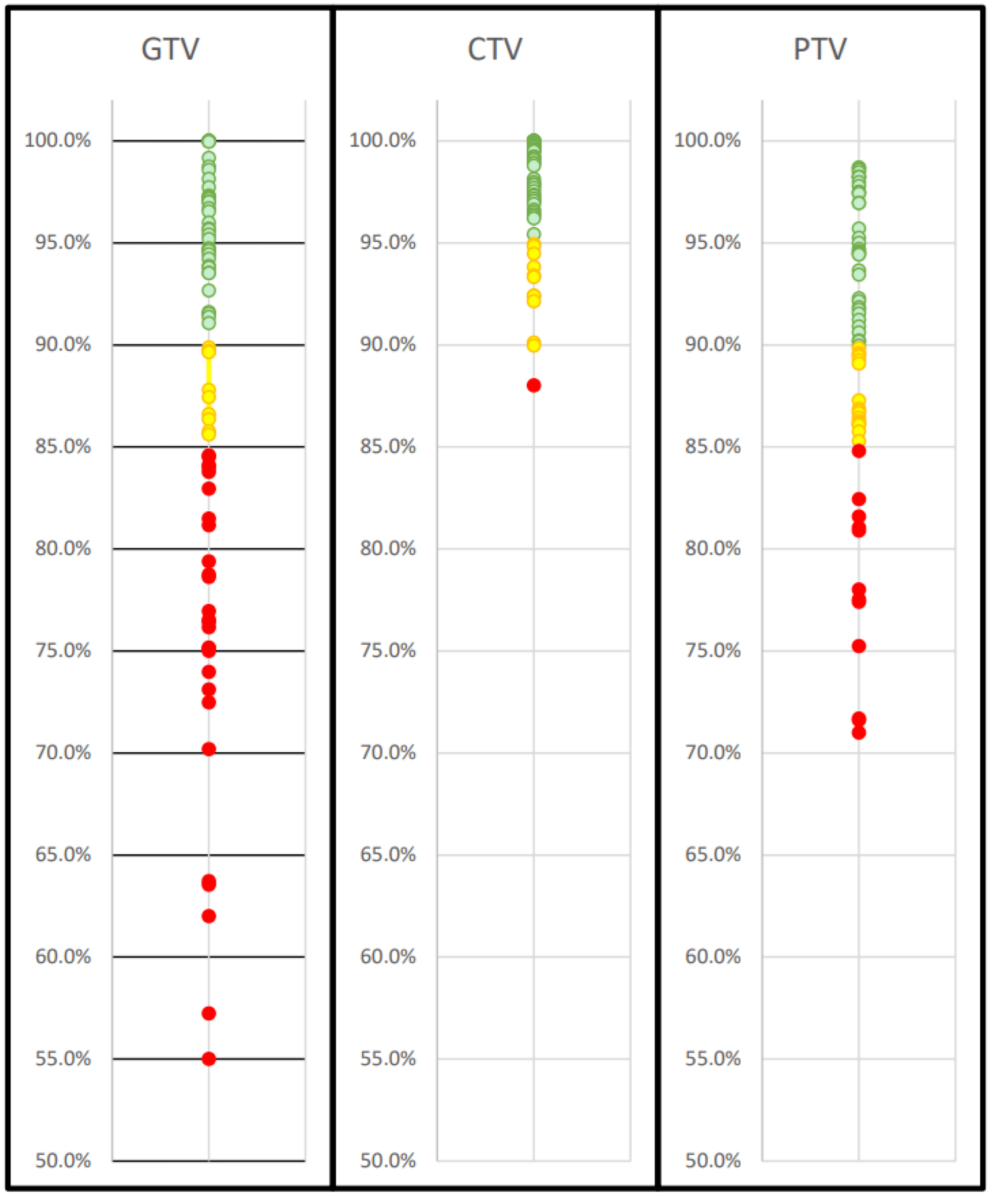
Summary of target coverage. Summary of target coverage using non-adaptive planning techniques. The GTV coverage exceeded 90% in 51% of fractions (green circles), was moderately covered in 12% (yellow circles) and poorly covered in 36% of fractions (red circles). The CTV coverage performed substantially better, with 81% of fractions achieving ≥95%, 17% achieving 90%-95% coverage, and only 2% falling below 90%. PTV coverage was >90% in 55% of fractions, moderately well covered for 27% of fractions, and poorly covered in 18% of fractions. All adaptive fractions achieved coverage all constraints for all targets; they are excluded from this figure as their results would all be clustered 95% or greater for all targets. CTV: clinical target volume, GTV: gross tumor volume, PTV: planning target volume.

For the non-adaptive fractions, 49% (36) had GTV coverage below 90% of the prescribed dose, while 12% (9) achieved moderately well coverage between 85% and 90%. A substantial number of fractions, 36% (27), had poor coverage below 85%, and a small subset 7% (5) showed very low coverage, falling below 70%. These GTV coverage results are summarized in Figure 1. Coverage failures were also analyzed per patient basis to identify any trends. Among the 13 patients, five demonstrated good GTV coverage, with either zero or one fraction out of five showing coverage below 90%. Four patients exhibited intermediate GTV coverage where either two or three of all five fractions failed to meet the 90% coverage threshold. Finally, four of the 13 patients exhibited poor GTV coverage, with four or five out of the five fractions failing to meet this metric.

CTV and PTV coverages were also evaluated for the non-adaptive plans. The CTV coverage was well maintained above 95% for 81% (52) of fractions and was moderately covered (90%-95%) for 17% (11) of fractions. Only 2% (one fraction) of the 64 investigated resulted in poor coverage below 90%, with 88% coverage. The PTV coverage was above 90% for 55% (35) of fractions and moderately covered (85%-90%) for 27% (17) of fractions. However, 18% (12) of fractions had poor coverage below 85%. These CTV and PTV coverage results are summarized in Figure 1. Conversely, the adaptive plans met coverage constraints for both CTV and PTV targets for all fractions.

For non-adaptive plans, the CTV coverage failures were also evaluated on a per-patient basis to identify trends. Ten of the 13 patients demonstrated good CTV coverage throughout their treatment course, with either zero or one fraction falling below 95% coverage; in fact, nine of these patients had no CTV coverage failures at all. Two of the 13 patients resulted in intermediate CTV coverage throughout their treatment course, with two or three of the five fractions falling below 95% coverage. Finally, only one patient showed consistently poor CTV coverage, with either four or five of the five fractions below 95% threshold. Again, for the adaptive treatment plans, the CTV coverage was maintained at a high level for all fractions studied. For the non-adaptive plans, the PTV coverage failures were also evaluated within each patient to identify trends. Six of the 13 patients resulted in good PTV coverage, with either zero or one fraction out of five falling below 90% coverage. Three of the 13 patients resulted in intermediate PTV coverage, with two or three of the five fractions falling below 90% coverage. Finally, four patients exhibited poor PTV coverage, where four or five of the five fractions were below the 90% threshold.

OAR constraints (anorectum and bladder)

**Figure 2:**
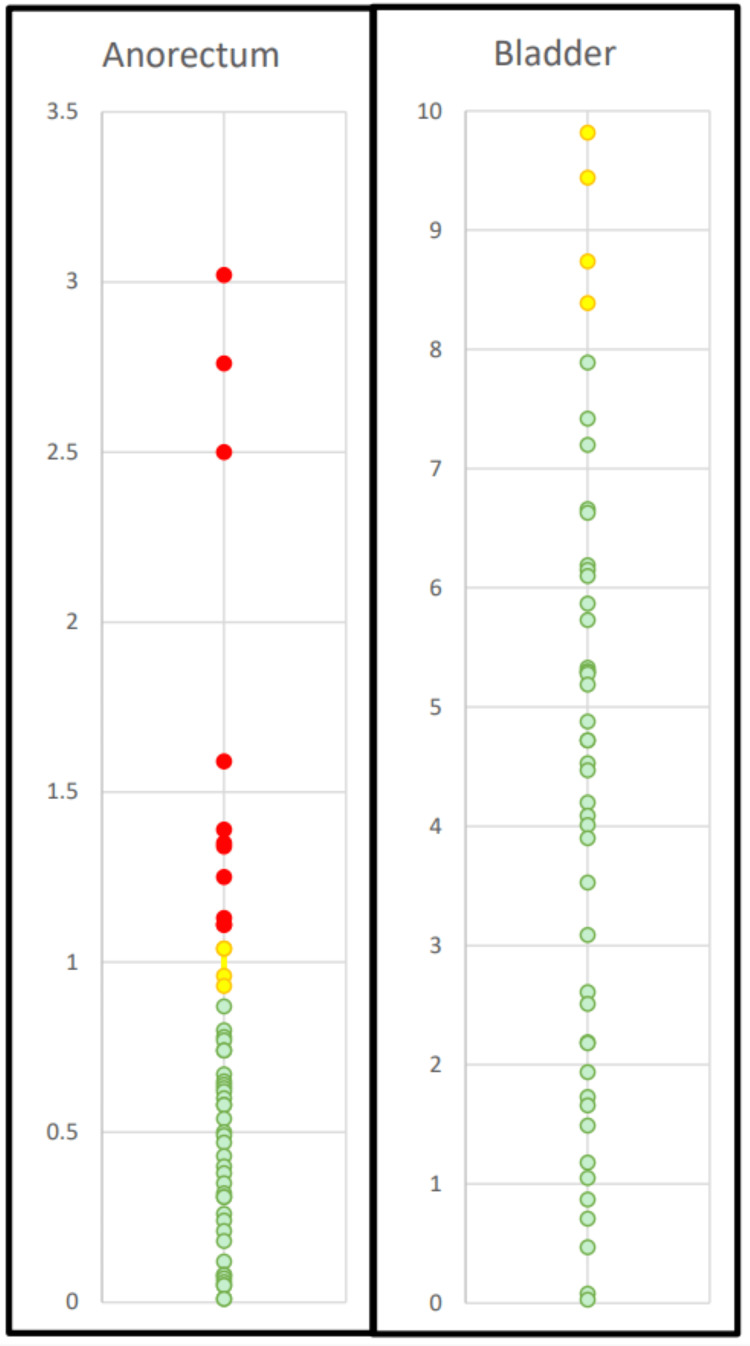
Summary of OAR sparing. Summary of OAR sparing using non-adaptive planning techniques. The anorectum constraint (<1 cm³ receiving ≥36 Gy) was met in 78% of fractions, with 22% of fractions failing this metric (red circles). Yellow circles represent fractions where the anorectum volume ranged between 0.9 and 1.1 cm^3^ at 36Gy, indicating values near the threshold. The green circles represent fractions where the anorectum volume was below 0.9 cm^3^ at 36Gy, indicating values significantly below the threshold. The bladder constraint (<10 cm^3^ receiving ≥36 Gy) was met in all fractions. In 9% of fractions (yellow circles), the bladder volume was close to the constraint, exceeding 8 cm^3^ at 36 Gy. All adaptive fractions achieved constraints for all OARs; they are excluded from this figure as their results would all be clustered together near the bottom of the figure. OAR: organ-at-risk.

The dosimetric differences between adaptive and non-adaptive fractions were compared for both the anorectum and the bladder. The adaptive plans performed exceptionally well due to the nature of the adaptive process; all 64 fractions in the adaptive cohort were reoptimized, thus ensuring that the anorectum and bladder constraints were met for all fractions studied. For the anorectum, the evaluation constraint was the volume receiving 36 Gy or higher; if more than 1 cm^3^ received ≥36 Gy, the constraint was considered failed. One of the 13 patients utilized a different anorectum constraint; therefore, the comparisons for the anorectum were not performed for that patient. Thus, a total of 59 fractions were analyzed. The anorectum showed notable differences between adaptive and non-adaptive fractions, where 78% (46) of non-adaptive fractions met the constraint, whereas none of the 59 adaptive fractions exceeded it. These results are summarized in Figure 2. Anorectum non-adaptive constraint failures were also evaluated on a per-patient basis to identify trends. Most patients exhibited good performance, with nine of the 12 patients having one or zero fractions that failed this constraint. Two patients had two fractions that failed the constraint, and one patient performed poorly, with all five non-adaptive fractions. Again, all adaptive fractions studied met anorectum constraints.

For the bladder, the evaluation constraint was the volume receiving 36 Gy or higher; if more than 10 cm^3^ received ≥36 Gy, the constraint was considered failed. Four of the 13 patients utilized different constraints; therefore, bladder comparisons were not performed for those patients. A total of 45 fractions were thus analyzed. The bladder demonstrated minimal differences between adaptive and non-adaptive fractions, with 100% (45) of non-adaptive fractions meeting the constraint and none of the adaptive fractions exceeding it. These bladder results are summarized in Figure 2.

DVH comparisons

**Figure 3:**
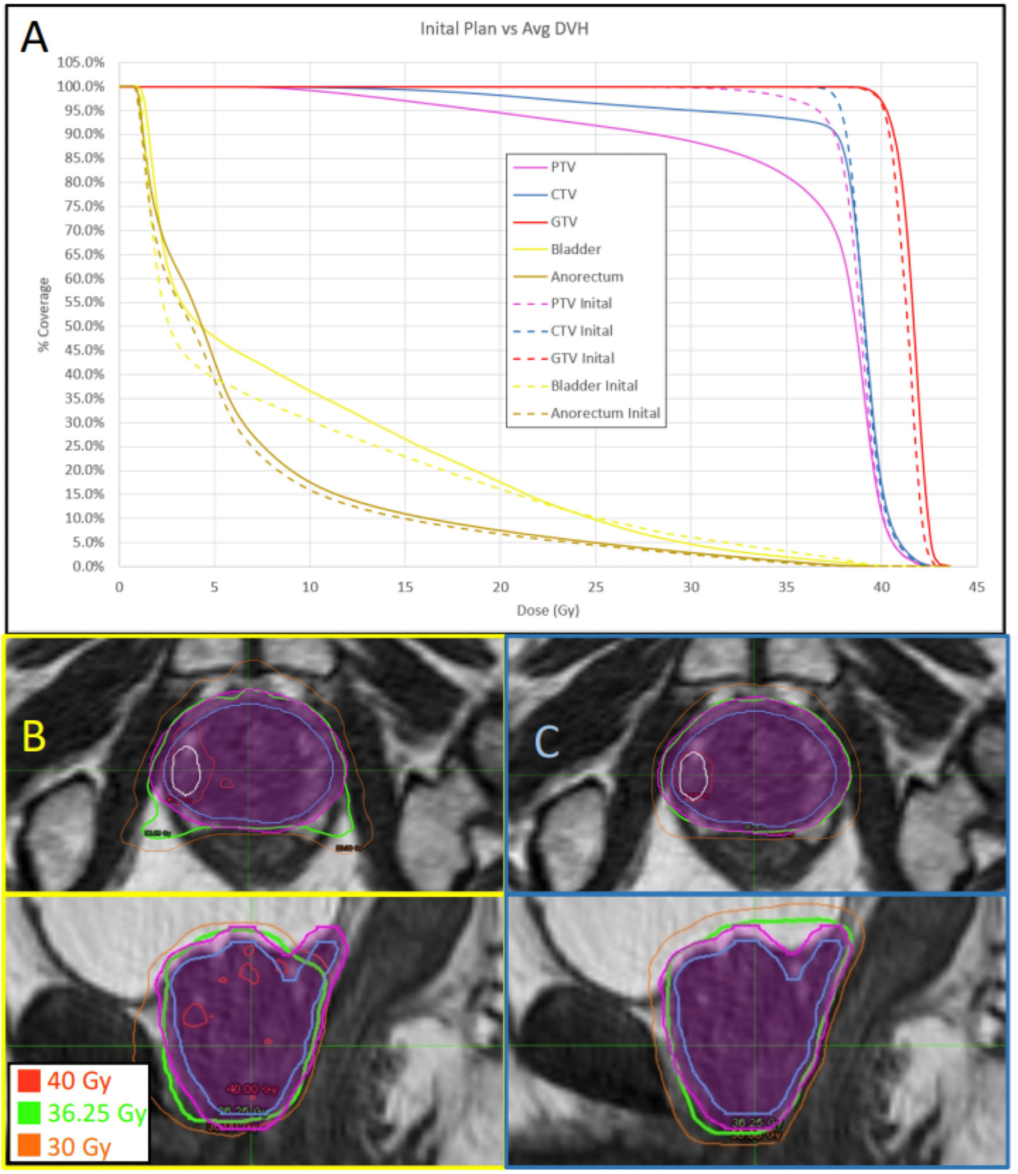
Example of CTV and PTV coverage deficiencies with non-adaptive techniques. Example of CTV and PTV coverage deficiencies using non-adaptive techniques. The DVH (A) from all five delivered non-adaptive fractions was averaged together from patient #1, and the CTV and PTV coverage percentage at the prescribed dose of 36.25 Gy was 93% and 78%, respectively (solid blue and solid magenta lines on the DVH). Contrasting this with the initial treatment plan's DVH (dashed lines), where the CTV coverage was 100% and the PTV coverage was 96%. All adaptive fractional plans passed coverage and OAR constraints and were similar to the initial DVH. The bottom panels demonstrate the non-adaptive and adaptive techniques for a single fraction in both axial and sagittal planes. With the non-adaptive shown in panel B (bordered in yellow) and the adaptive shown in panel C (bordered in blue). The PTV is the magenta contour, the CTV is the blue contour, and the GTV is the white contour. Note the superior conformality of the prescription isodose line to the CTV and PTV structures in panel C. DVH: dose volume histogram, OAR: organ-at-risk, CTV: clinical target volume, GTV: gross tumor volume, PTV: planning target volume.

**Figure 4:**
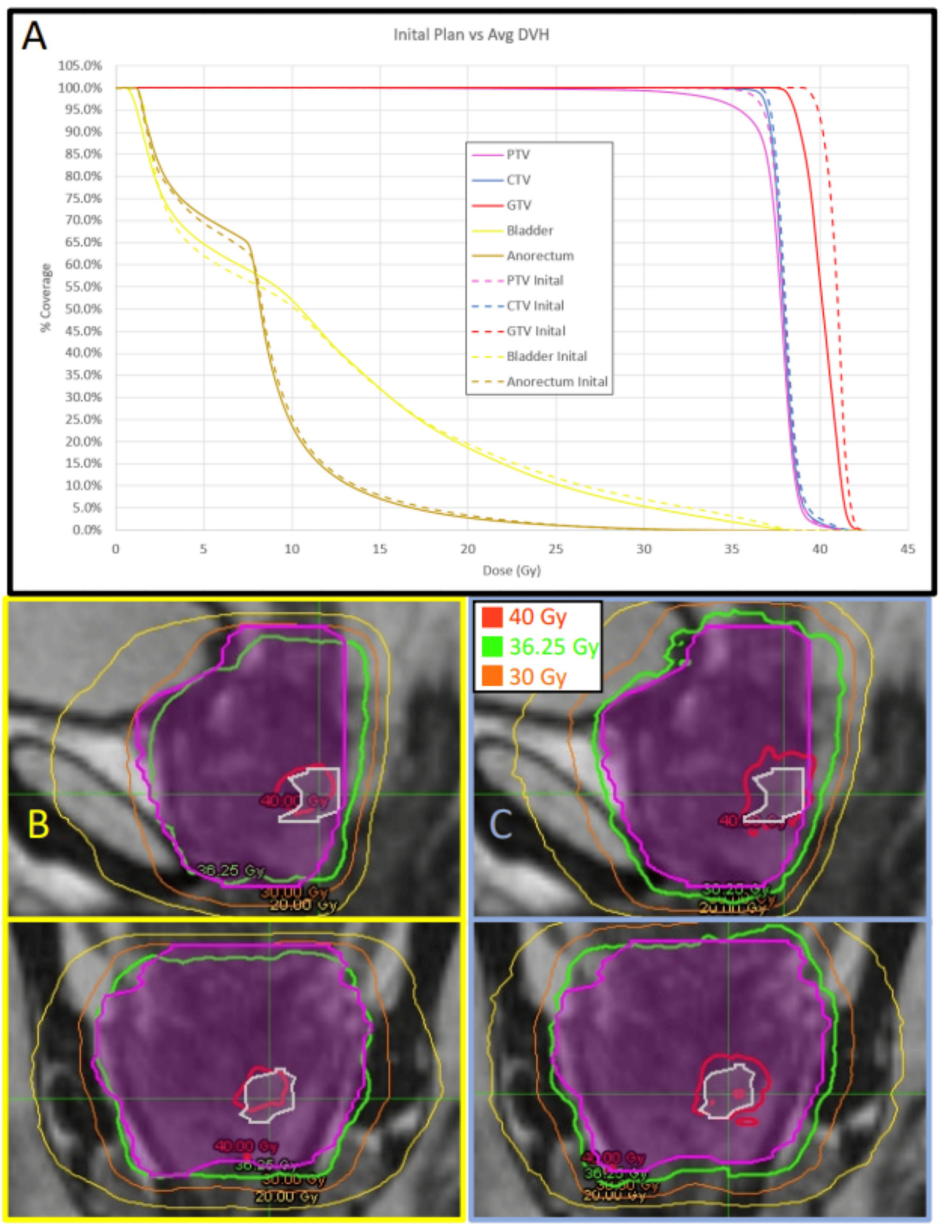
Example of GTV coverage deficiency using non-adaptive techniques. Example of GTV coverage deficiency using non-adaptive techniques. The DVH in Panel A represents the average of all five delivered non-adaptive fractions for patient #2, showing a GTV coverage of 57% at the prescribed dose of 40 Gy (solid red line). Contrasting this with the initial treatment plan’s DVH (dashed lines), where the GTV coverage was 90%. All adaptive fractional plans passed coverage and OAR constraints and were similar to the initial DVH. The bottom panels demonstrate the non-adaptive and adaptive techniques for a single fraction in both axial and sagittal planes. With the non-adaptive shown in panel B (bordered in yellow) and the adaptive shown in panel C (bordered in blue). The PTV is the magenta contour and the GTV is the white contour. Note the superior conformality of the prescription isodose line to the GTV structure in panel C. DVH: dose volume histogram, OAR: organ-at-risk, CTV: clinical target volume, GTV: gross tumor volume, PTV: planning target volume.

To assess the potential differences between adaptive and non-adaptive planning over the entire treatment course, not just on a fraction-by-fraction basis, DVHs from all fractions were accumulated over the course for each technique and patient. Several patients demonstrated significant improvements in CTV and PTV coverage. Figures 3A-3C provide an example where the non-adaptive fractions showed inferior CTV and PTV coverage at the prescribed dose (93% and 78%, respectively), while the adaptive fractions consistently met the prescribed coverage constraints. Similarly, several patients showed significant gains in GTV coverage. Figures 4A-4C provide an example where non-adaptive fractions had poor GTV coverage (57%), whereas adaptive fractions achieved the prescribed target coverage.

**Figure 5:**
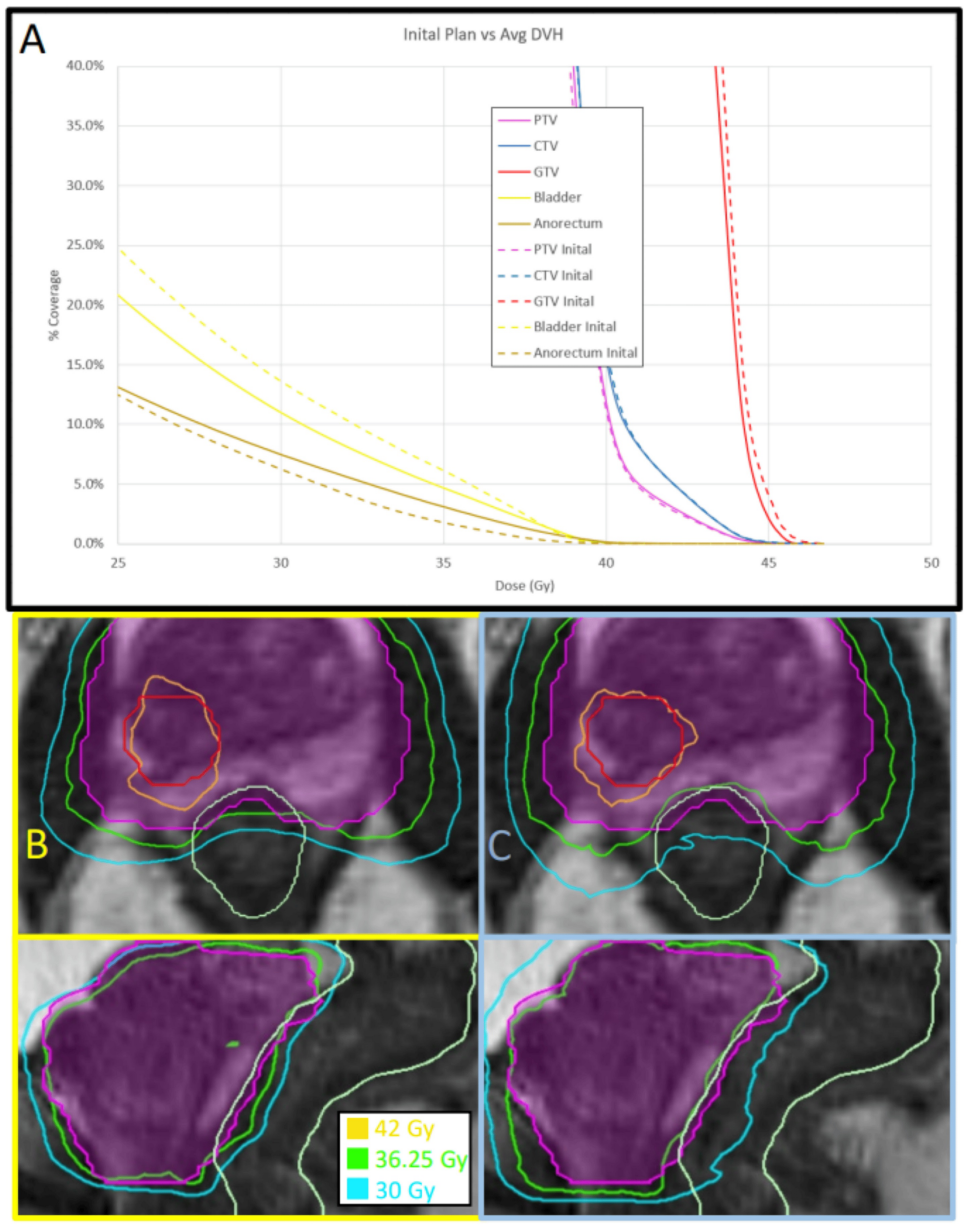
Example of anorectum sparing deficiency using non-adaptive techniques. Example of anorectum sparing deficiency using non-adaptive techniques. Panel A shows the averaged DVH from all five delivered non-adaptive fractions for patient #11. The anorectum constraint (<1 cm³ receiving ≥36 Gy) was violated, with 1.7 cm^3^ at 36 Gy (solid brown line on the DVH), exceeding the prescribed limit. Contrasting this with the initial treatment plan’s DVH (dashed lines), where the anorectum had 0.9 cm^3^ at 36 Gy. All adaptive fractional plans passed coverage and OAR constraints and were similar to the initial DVH. The bottom panels demonstrate the non-adaptive and adaptive techniques for a single fraction in both axial and sagittal planes. With the non-adaptive plan shown in panel B (bordered in yellow) and the adaptive plan shown in panel C (bordered in blue). The PTV is the magenta contour, the GTV is the red contour, and the anorectum is the light green contour. Note the prescription isodose line of 36.25 Gy (bright green) extending into the anorectum contour in panel B but essentially conforming to the edge of the anorectum in panel C. DVH: dose volume histogram, OAR: organ-at-risk, CTV: clinical target volume, GTV: gross tumor volume, PTV: planning target volume.

Several patients showed dramatic differences in anorectum sparing between the adaptive and non-adaptive strategies, as shown in Figures 5A-5C. Patient #11 exhibited the most significant anorectum sparing: the prescription requires that less than 1 cm^3^ of the anorectum receive 36 Gy. The non-adaptive approach resulted in an average of 1.7 cm^3^ (1.11 cm^3^ to 3.02 cm^3^) receiving 36 Gy, whereas the adaptive approach met the constraint in all fractions, with an average of 0.17 cm^3^ (0.13 cm^3^ to 0.21 cm^3^). In contrast, bladder sparing differences were minimal, with all non-adaptive fractions meeting the prescribed constraint.

IDL comparisons (single fraction comparisons)

To facilitate the visualization of the potential benefits of the adaptive process, isodose lines from selected fractions were captured to compare the adaptive and non-adaptive approaches. Figure 3 demonstrates the increased CTV and PTV coverage of the adaptive approach as compared to the non-adaptive for one patient. The adaptive plan achieved the prescribed PTV coverage of 95.5% of the PTV receiving 36.25 Gy, compared to 77.5% coverage by the non-adaptive plan. Similarly, the CTV coverage achieved 100% for the adaptive plan versus 92.4% for the non-adaptive plan. Figure 4 demonstrates the improved coverage achieved with adaptive planning techniques, where the adaptive plan achieved the prescribed 90% coverage at 40 Gy, compared to only 49% coverage achieved by the non-adaptive plan. Figure 5 shows the potential sparing of the anorectum with the adaptive plan, which delivered 36 Gy to only 0.13 cm^3^ of volume, compared to 3.02 cm^3^ with the non-adaptive plan. This OAR’s tolerance was set at a maximum of 1.0 cm^3^ receiving 36 Gy.

## Discussion

This analysis evaluated the potential dosimetric benefits of adaptive strategies for prostate SBRT using a simultaneous integrated boost technique under MRI guidance. Historically, delivering precise radiation therapy to these targets has been challenging due to inter- and intra-fraction prostate motion, filling of the bladder and rectum, combined with the difficulty in accurately determining intraprostatic targets using on-board kV X-ray imaging. The treatment paradigm offered by MRgART has the potential to overcome these challenges by maintaining the target coverage at the prescribed dose while meeting OAR tolerances. Additionally, by combining the image fidelity of MRI with daily optimization, the uncertainty in delivered dose may be greatly reduced.

The most consistent advantage of daily adaptive planning was its ability to maintain the prescribed GTV coverage compared to non-adaptive techniques. Non-adaptive plans often demonstrated suboptimal coverage, with approximately half of the studied fractions achieving less than 90% coverage at the prescribed dose, and around 7% of fractions falling below 70% coverage. In contrast, adaptive treatments resulted in GTV coverage at the prescribed dose because the re-optimized plans are normalized to achieve this coverage. In the adaptive setting, the main source of uncertainty arises from the day-to-day variability in the contouring of the GTV target. Given this source of uncertainty perhaps margins for adaptive plans may be appropriate, although likely smaller as compared to non-adaptive plans. Target coverage in the non-adaptive setting may be suboptimal for several reasons, with positional uncertainties and morphological changes being the main culprits. These potential causes of suboptimal coverage are accounted for in the adaptive setting allowing for consistent coverage throughout the treatment course. The benefits of the adaptive strategy for PTV and CTV coverage are less pronounced compared to those observed for the GTV, as treatment sessions for some patients exhibit consistent challenges in adequately covering these targets regardless of planning strategy.

There have been different studies supporting either adaptive or non-adaptive MRgRT for prostate cancer treatments. In a study by Sandoval et al., 34 patients with favorable and unfavorable intermediate-risk prostate cancer underwent MR-guided SBRT without adaptation on the 0.35T MRIdian System [29]. They assessed tumor control based on PSA levels and American Urological Association (AHU) scores over follow-ups up to ~20 months and concluded that non-adaptive SBRT treatments are successful with manageable toxicity with no need for adaptation, reducing the overall treatment time. In their study, the CTV included the prostate and seminal vesicles, treated to 36.25 Gy over five fractions. In a related study, Teunissen et al. investigated adaptive ultra-hypofractionated prostate cancer treatments (5 × 7.25 Gy to the PTV) of 425 patients on the 1.5T MR-linac and assessed the toxicity of the OARs with follow-up of 12 months [30]. Their study found the treatments to be safe and effective, with the peak of genitourinary and gastrointestinal toxicities being reported at three months. While these two studies reported the effectiveness of the adaptive and non-adaptive treatments, neither of them had a control arm to compare the results to the opposite group and they did not assess the target coverage. Also, the treatments in these studies did not include a boost volume to investigate the benefit of adaptation in these targets.

Adaptive planning also has the potential to better spare OAR structures as well. In this study, some improvement in rectal sparing was observed using adaptive strategies, while bladder sparing was effective using both techniques. Failures in anorectum sparing were primarily concentrated in a small subset of patients, with most patients achieving effective sparing even with non-adaptive techniques. These findings suggest that, in the patient population studied, the main benefit of adaptive planning is not OAR sparing but in target coverage.

Like many adaptive studies, this work has several limitations. The study included 13 patients and a total of 64 fractions. While not ideal, this is a sufficient sample size to draw meaningful conclusions. Another limitation is the lack of complete consistency in the constraints and the prescribed coverage percentages; although most of the patients had consistent metrics, there were some deviations. Despite these variations, patients with differing constraints were included because we accounted for discrepancies by reporting the number of constraint violations, an approach often necessary in clinical science. Another limitation to this study is related to the simulated nature of the adaptive treatments; bladder and rectal morphological changes related to filling or anatomical movement during the treatment planning process are not possible to capture with this method but can have an impact on treatment delivery in non-simulated adaptive treatments. Many methods exist to reduce the impact of these changes on the delivery outcome and to evaluate whether meaningful changes have occurred prior to beginning treatment delivery, i.e., re-imaging prior to commencing treatment to ensure only trivial anatomical changes have occurred as well as the use of real-time imaging during treatment to evaluate changes. We feel that the effect of these potential changes on the delivered dose distribution will be minimal as compared to the planned dose distribution if best practices for adaptive treatment workflows are implemented.

Another analysis method we considered but ultimately did not include was deformable accumulation of fractional doses back to the original planning dataset. This approach could have provided better insights into the impact of cumulative fractional changes over the entire treatment course on a single dataset. However, we chose not to pursue this strategy due to the inherent uncertainties in deformable image registration algorithms, which exceeds the necessary accuracy requirements for this study. Given the steep dose gradients in these SBRT treatments, even small errors on the order of 1-2 mm during dose accumulation can lead to significant inaccuracies; therefore, this strategy was not employed.

Finally, contouring accuracy remains a limitation in both adaptive and non-adaptive studies. To minimize this uncertainty, our study followed best practices by involving experienced radiation oncologists specialized in prostate cancer treatments to contour both the initial treatment plans and the simulated adaptive fractions. Furthermore, when the adaptive treatments were simulated, there were no time constraints on contouring, allowing for careful and precise delineation. Nevertheless, despite these measures, some degree of contouring uncertainty is inevitable in all dosimetric studies.

## Conclusions

The main takeaway from this work is that the necessity of adaptive planning for MRI-guided SBRT in prostate cancer remains uncertain and is likely patient-specific. Key considerations include whether a simultaneous boost is planned for the dominant intraprostatic lesion and the margin strategy employed for these targets. If a minimal margin approach is used for the PTV and GTV, adaptive planning may have a greater impact on maintaining target coverage and potentially improving patient outcomes. On the other hand, if strict bowel and bladder preparation protocols are consistently followed throughout the treatment course and no boost to dominant lesions is planned, the added value of adaptive planning may be more limited. Some patients exhibit less reproducible anatomy, often due to inconsistent bowel and bladder preparation, and may therefore derive greater benefit from adaptive strategies. A practical approach may be to implement a monitoring protocol to identify such patients early in their treatment course, allowing for a timely transition to adaptive planning when appropriate.
